# Evening light environments can be designed to consolidate and increase the duration of REM-sleep

**DOI:** 10.1038/s41598-022-12408-w

**Published:** 2022-05-24

**Authors:** Daniel Vethe, H. J. Drews, J. Scott, M. Engstrøm, H. S. A. Heglum, J. Grønli, J. P. Wisor, T. Sand, S. Lydersen, K. Kjørstad, P. M. P. Faaland, C. L. Vestergaard, K. Langsrud, H. Kallestad

**Affiliations:** 1 Department of Mental Health, Norwegian University of Science and Technology, Trondheim, Norway; 2 Division of Mental Health Care, St. Olav’s University Hospital, Trondheim, Norway; 3 Institute of Neuroscience, University of Newcastle, Newcastle, UK; 4 Department of Neuromedicine and Movement Science, Norwegian University of Science and Technology, Trondheim, Norway; 5 Department of Clinical Neurophysiology, St. Olav’s University Hospital, Trondheim, Norway; 6 Novelda AS, Trondheim, Norway; 7 Department of Biological and Medical Psychology, University of Bergen, Bergen, Norway; 8 Sleep and Performance Research Center and Department of Translational Medicine and Physiology, Elson S. Floyd College of Medicine, Washington State University, Spokane, WA USA; 9 Regional Centre for Child and Youth Mental Health and Child Welfare, Department of Mental Health, Norwegian University of Science and Technology, Trondheim, Norway

**Keywords:** Translational research, Physiology, Medical research

## Abstract

Evening exposure to short-wavelength light has disruptive effects on circadian rhythms and sleep. These effects can be mitigated by blocking short-wavelength (blue) frequencies, which has led to the development of evening blue-depleted light environments (BDLEs). We have previously reported that residing 5 days in an evening BDLE, compared with residing in a normal indoor light environment of similar photopic lux, advances circadian rhythms and increases the duration of rapid eye movement (REM) sleep in a randomized cross-over trial with twelve healthy participants. The current study extends these findings by testing whether residing in the evening BDLE affects the consolidation and microstructure of REM sleep in the same sample. Evening BDLE significantly reduces the fragmentation of REM sleep (*p* = 0.0003), and REM sleep microarousals in (*p* = 0.0493) without significantly changing REM density or the latency to first REM sleep episode. Moreover, the increased accumulation of REM sleep is not at the expense of NREM stage 3 sleep. BDLE further has a unique effect on REM sleep fragmentation (*p* = 0.0479) over and above that of circadian rhythms phase-shift, indicating a non-circadian effect of BDLE. If these effects can be replicated in clinical populations, this may have a therapeutic potential in disorders characterized by fragmented REM sleep.

## Introduction

Artificial light is an important aid to visual acuity in the evening and at nighttime. This light exposure also has extensive non-image-forming (NIF) effects on human sleep and circadian rhythms^1–5^. These effects are primarily driven by intrinsically photosensitive retinal ganglion cells (ipRGCs) that both entrain the central circadian pacemaker to the light environment and directly influence sleep^6^. The photosensitivity of ipRGCs is predominantly in the short-wavelength blue light range (λ_max_ ≈ 480 nm). As such, there is an opportunity to design artificial evening light environments with less impact on these systems. This has led to the development of acute hospital facilities that utilize an evening blue-depleted light environment (BDLE) in order to improve sleep and circadian rhythmicity for inpatients^7^. In a previous publication^8^, we reported findings from a pilot study undertaken with 12 healthy participants who resided in a new psychiatric unit prior to its official opening and who were exposed to both an evening BDLE and a standard light environment (standard LE) for 5 days each. The study demonstrated that, in addition to a phase-advancing effect on circadian rhythms and an increase in sleep duration, there was also a significant increase in the duration of rapid eye movement (REM) sleep. Noting that REM sleep is a heterogenous sleep stage^9^, with alternations between active (phasic) and comparatively quiescent (tonic) substages, the downstream effects of increased duration might depend on REM sleep fragmentation and microstructure.

In adults, REM-sleep normally occupies 20–25% of the total sleep time and supports several functions such as brain temperature regulation, synaptic plasticity, and complex cognitive processes such as memory consolidation. Notably, research indicates that REM sleep, and particularly phasic REM sleep, plays a role in the overnight processing of emotionally salient information and thus emotion regulation^9–13^. Therefore, we can hypothesize that an increase in the duration of REM sleep after exposure to a BDLE might have many potential benefits for individuals. However, recent findings in insomnia suggest that the degree of REM sleep fragmentation modulates the favorable effect of REM sleep on amygdala reactivity^14–16^. This “restless” REM-sleep may then increase emotional distress and theoretically be a risk factor for insomnia and mental illness^15^. In the case of depression, increased REM sleep duration in combination with shorter latency to first REM sleep episode and increased REM-density is common, and is understood as a maladaptive disinhibition of REM-sleep that increases vulnerability and impacts treatment responses^10,11,17–23^. Although this research has mostly been undertaken in clinical populations, these characteristics of REM sleep might offer indications whether the increased REM sleep duration is adaptive or dysfunctional. Thus, they represent useful elements to explore in the context of evening light and its effects on REM sleep.

There are at least two conceivable mechanisms by which evening light exposure may have effects on REM sleep. First, REM sleep regulation is tied to circadian rhythms^24^, with increased propensity to enter REM sleep in the late night/early biological morning. A phase-advance of circadian rhythms, as we and others have reported for evening BDLE^8,25^, may result in more REM sleep earlier in the night, possibly at the expense of slow-wave sleep (SWS; N3) that has higher propensity in the first sleep cycles, and also is associated with numerous physiological health benefits^26^. Second, artificial evening light exposure results in higher arousal and alertness before bedtime^2,27,28^, and may reduce slow wave activity (0.75–4 Hz) in the first sleep cycle^6^. This suggests a way by which arousal may be carried into the sleep period, possibly also altering REM sleep fragmentation or microstructure. Moreover, in the case of insomnia, pre-bedtime hyperarousal, has been linked to fragmented REM sleep. We and others have found that evening blue-depleted light diminishes pre-bedtime arousal^25^. This represents a non-circadian pathway for the effects of evening light exposure on REM sleep. Due to REM sleep being associated to both circadian and arousal processes, distinguishing the mechanisms by which evening light may modify REM sleep is a challenge.

In summary, we previously reported a significant increase in in REM sleep duration when healthy participants resided in an evening BDLE^8^. In this study, we explore this finding in more detail by first examining whether there are any differential effects of residing in an evening BDLE compared with a standard LE on REM sleep fragmentation and REM-density. Second, to explore potential mechanisms of change, we test if changes in REM sleep parameters are associated with phase-shift of circadian rhythms. Finally, we examine the temporal dynamic of the accumulated REM sleep, and contrast that with the accumulation of non-REM stage 3 sleep (N3).

## Results

The sample of healthy participants consisted of 7 women and 5 men. See Table 1 for baseline descriptive statistics. Participants had a similar number of sleep cycles with 4.3 cycles (95% CI 3.9–4.7) in the BDLE and 4.3 cycles (95% CI 3.9–4.8) in the standard LE. The mean duration of sleep cycles was also similar in both conditions with 103.6 min (95% CI 94.4–113.2) in the BDLE and 103.6 min (95% CI 94.5–113.3) in the standard LE. For further descriptive information regarding REM sleep cycles, REM fragmentation, and REM microarousals see Supplementary Figs. S1, S2, S3, S4, S5, and S6.

**Table 1:** Baseline sleep and circadian rhythm characteristics of the sample (N = 12).

	Mean	SD	Range
Age	23.0	3.1	20–28
Baseline DLMO	21:11	0:37	19:59–21:57
Baseline bedtime	23:54	0:26	23:09–00:36
Baseline rise-time	08:22	0:29	07:47–09:03
Baseline total sleep time	08:03	0:28	07:26–08:54

N = 12 participants for all variables and analyses.

Sample means, standard deviations and ranges of baseline characteristics. Rise-times, bed-times and total sleep time values are reported as sample mean values, standard deviations and ranges of the underlying individual participants’ mean, over the seven days prior to randomization (days − 7 to − 1). Baseline DLMO refers to the DLMO-assessment undertaken on day 1 of the study. *DLMO* Dim Light Melatonin Onset.

### REM-sleep

As reported previously^8^, individuals demonstrated a significantly longer REM-sleep duration (see Table 2) when residing in the evening BDLE (89.7 min, 95% CI 80.8–98.6) compared with the standard LE condition (75.8, 95% CI 66.9–84.7)^8^.

**Table 2:** Effects of phase shift and light environment on REM sleep duration, REM sleep fragmentation, REM sleep microarousals, and REM-density.

Step	REM sleep duration			REM sleep fragmentation			REM sleep arousals			REM-density		
	Estimate	95% CI	*p*	Estimate	95% CI	*p*	Estimate	95% CI	*p*	Estimate	95% CI	*p*
**1**												
BDLE	13.88	5.82 to 21.88	0.0018*	− 3.71	− 6.79 to − 1.50	0.0003*	− 12.58	− 25.38 to − 0.04	0.0493*	0.007	− 0.011 to 0.025	0.432
Phase shift	− 22.9	− 35.23 to − 10.55	0.0007*	4.56	1.00 to 9.86	0.0092*	15.11	− 2.22 to 32.96	0.089	0.005	− 0.025 to 0.034	0.757
**2**												
BDLE	6.65	− 4.95 to 18.24	0.27	− 3.68	− 7.87 to − 0.02	0.0479*	− 10.34	− 26.24 to 6.32	0.22	0.024	− 0.003 to 0.051	0.090
Phase shift	− 15.21	− 33.25 to 2.83	0.11	0.06	− 5.79 to 6.80	0.99	4.74	− 18.71 to 25.73	0.68	0.036	− 0.009 to 0.080	0.126

Results from four linear mixed models with total REM sleep duration, REM sleep fragmentation, REM sleep microarousals, or REM-density as dependent variable and participant ID as random effect. The models are fitted in two steps. In step I, BDLE and Phase shift are included separately as covariates, and in Step II, they are included simultaneously. The estimates, 95% confidence intervals, and *p*-values were calculated from mixed models with N = 12 participants. BDLE = Blue-depleted light environment. * = significant effect of BDLE or phase shift, at *P* < 0.05.

**Figure 1:**
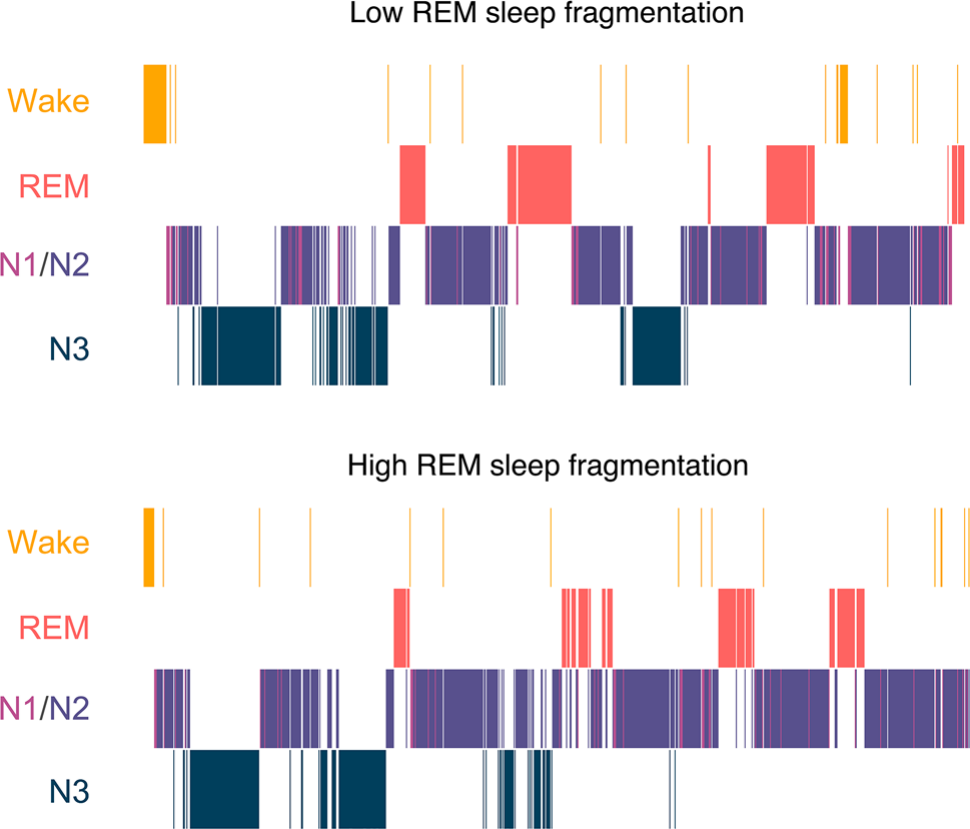
Example hypnograms of two nights with either low REM sleep fragmentation or high REM sleep fragmentation. Both examples are from the same individual with the low REM sleep fragmentation being in the BDLE condition and the high REM sleep fragmentation being in the standard LE condition.

As shown in Table 2, further analyses demonstrate that individuals show less REM-sleep fragmentation in the evening BDLE (6.6%, 95% CI 3.8, 10.1) compared with in the standard LE condition (10.3%, 95% CI 7.7–14.2) (see Fig. 1). Moreover, there was also a reduction in cortical microarousals during REM sleep in the BDLE with 24.6 arousals (95% CI 15.6–35.4) compared with 37.2 arousals (95% CI 28.0–48.2) in the standard LE. There was no significant difference in REM-density between the conditions.

### Associations between changes in REM-variables and the phase-shift of dim light melatonin onset (DLMO)

As shown in Table 2, phase advancement of the DLMO was associated with longer duration of REM-sleep and reduced REM-fragmentation (see Supplementary Fig. S7). When BDLE and phase-shift were simultaneously entered in the second step, there were no unique associations between either construct and REM-sleep duration. However, there was a unique effect of BDLE over and above that of phase-shift for REM-fragmentation (see Table 2).

### Post hoc analyses by condition order

As reported in the previous publication^8^, there was an effect of order of exposure to the two conditions on phase-shift of DLMO. Given the association of circadian rhythms and REM sleep it was considered relevant to also test for order-effects in the current REM-outcome variables. The effect of BDLE on REM sleep fragmentation and REM sleep arousal was only significant in Period 1. However, there was no significant order effect of condition on any of the REM sleep variables (see Table 3).

**Table 3:** Effects of BDLE on REM sleep outcomes depending on whether BDLE is given in period 1 or period 2, and the effect of order on the BDLE-effect.

	BDLE in period 1			BDLE in period 2			Order effect		
	Estimate	95% CI	*P*	Estimate	95% CI	*P*	Estimate	95% CI	*P*
REM duration	5.3	− 13.5 to 24.2	0.56	22.4	3.5 to 41.2	0.024*	17.0	− 17.7 to 51.8	0.32
REM fragmentation	− 5.9	− 13.2 to − 0.4	0.036*	− 1.5	− 7.7 to 5.0	0.66	− 4.5	− 16.8 to 6.3	0.46
REM sleep microarousals	− 18.66	− 39.09 to 0.61	0.058	− 6.45	− 26.9 to 13.4	0.51	12.22	− 18.4 to 43.1	0.428
REM density	0.002	− 0.05 to 0.05	0.96	0.01	− 0.04 to 0.07	0.62	0.01	− 0.08 to 0.12	0.82

Results from three linear mixed models with total REM sleep duration, REM sleep fragmentation, REM sleep microarousals, or REM-density as dependent variable and participant ID as random effect. The order effects are reported as the interaction between period and condition. N = 12 participants. BDLE = Blue-depleted light environment. * = significant effect at *P* < 0.05.

### Accumulation of REM-sleep and N3

**Figure 2:**
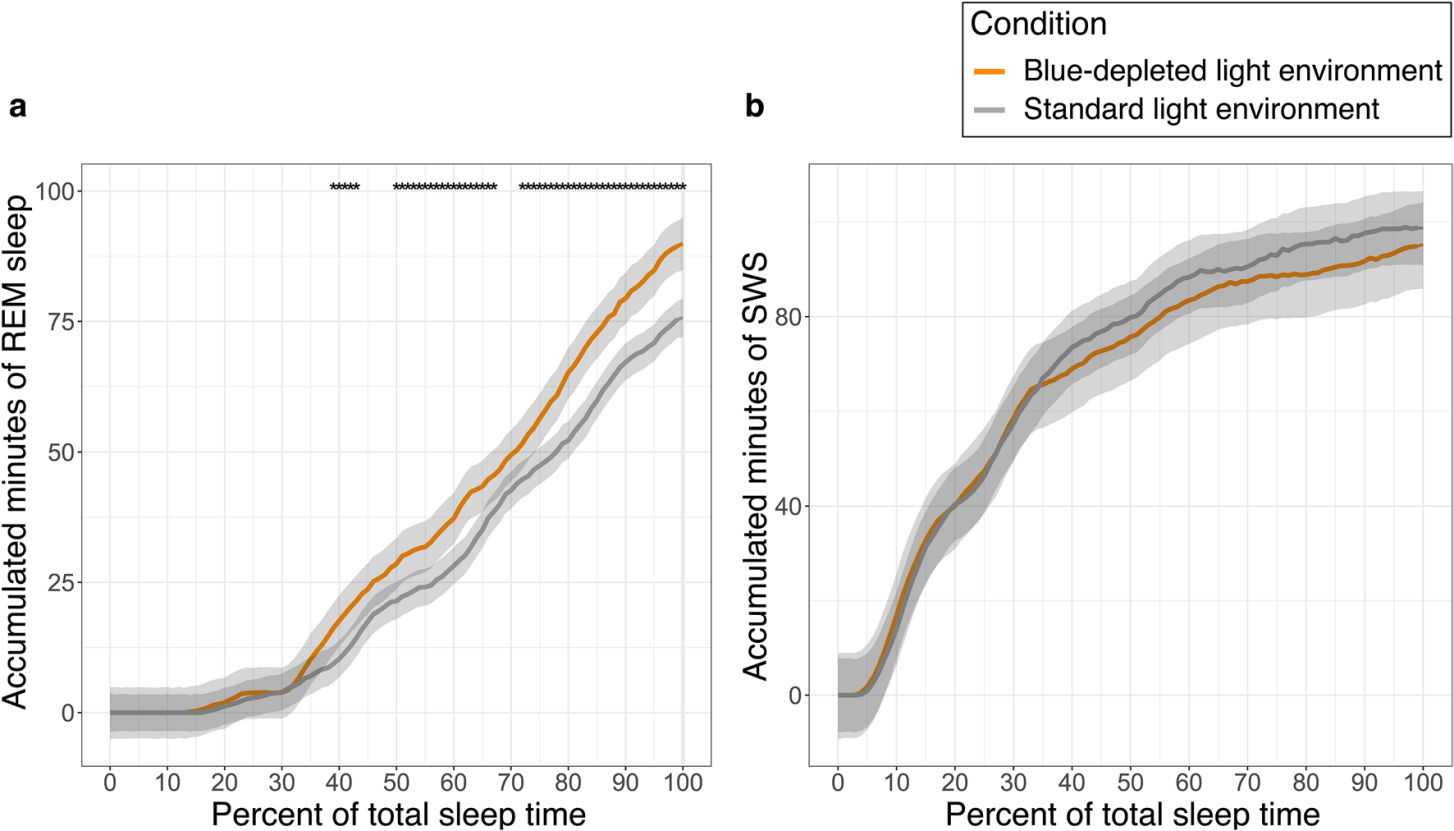
Average accumulated minutes in (**a**) REM sleep and (**b**) slow-wave sleep by percentage of the total sleep time. 95% confidence intervals, and *p*-values were calculated from a mixed model with N = 12 participants. Accumulated minutes as dependent variable, percent of total sleep time as a 101 level factor and light environment and their interaction as covariates, and participant ID as random effect. N = 12 participants. The *p*-values are Bonferroni-corrected for 101 comparisons.

Time in REM-sleep accumulated more rapidly when individuals were residing in the evening BDLE compared with the standard LE. As shown in Fig. 2a, the difference between conditions reached statistical significance from about 40% of the total sleep time onwards, increasing in magnitude after about 75% of total sleep time until the end of the sleep period. There were no significant differences between LE conditions in the accumulation of N3 sleep (see Fig. 2b).

## Discussion

In our previous study we reported a phase-advance of circadian rhythms and an increase in REM sleep duration when residing in an evening BDLE^8^. The current study extends these findings by showing that residing in an evening BDLE also a reduces the fragmentation of REM sleep and the number of cortical microarousals during REM sleep in the same sample. However, REM-density did not change after exposure to an evening BDLE. This suggests that the duration of REM sleep was longer, more consolidated, and with fewer microarousals, but that its microstructure, in terms of the balance between phasic and tonic REM sleep, remained unchanged in these healthy individuals. To the best of our knowledge, this is the first demonstration of an intervention based on light exposure affecting the consolidation of REM sleep.

REM-sleep follows a circadian rhythm with higher propensity to enter REM sleep in the second half of the night^24^. Therefore, a further aim of this study was to explore if any changes in REM sleep variables were associated with the phase-shift of DLMO. Although we did find this for the duration of REM sleep, the effect of phase-shift could not be distinguished from that of BDLE. It is known that, evening administration of melatonin induces a phase-advance of circadian rhythms and increase REM sleep duration, most prominently in the first sleep-cycle^29^. Conversely, evening exposure to short-wavelength light phase-delays circadian rhythms and reduces REM-sleep duration^2^, especially in the first REM sleep episode^6^. In the current study, it can be hypothesized that the effect of BDLE on REM sleep duration may be mediated through a phase-advance of circadian rhythms. Moreover, we have previously reported that the phase-advance was about 30 min larger in BDLE compared with standard LE, which roughly corresponds to the increased accumulation of REM-sleep starting about 30 min earlier in BDLE. One previous study has found that increased SWS accumulation may be at the expense of REM sleep after different light exposures^30^. The faster accumulation of REM-sleep after BDLE in our study, does not seem to be at the expense of N3 sleep.

Similar to the duration of REM sleep, we also found an effect of phase-shift on REM sleep fragmentation. However, BDLE had an additional, albeit marginally significant effect over and above the effect of phase-shift, explaining nearly all the variance in the effect of phase-shift. This may suggest that there is a non-circadian effect of evening light exposure on REM sleep fragmentation. Moreover, in the previous publication^8^ we found that the effect of BDLE on phase-shift was stronger in period 2 of the study. In the current study, we find in contrast that the effects of BDLE on REM sleep fragmentation were stronger in period 1, although there was no significant order effect. This further supports the notion that there may be additional non-circadian driven effects of BDLE. Interestingly, recent studies both with animal models and in humans have identified non-circadian pathways by which light can exert direct NIF-effects^31,32^. A non-circadian effect could potentially be mediated by lower arousal as an effect of the BDLE. Increased arousal has indeed been found after evening short-wavelength light exposure^2,27,33^, and this effect seems to be carried over into the sleep period^2,6,33^. On the contrary, lower levels of wake EEG-derived alertness has been observed after BDLE^25^, and we also found reduced neurocognitive arousal in our previous report on this sample^8^. In the current study we found a trend toward less microarousals from NREM sleep (see supplementary Table S1). Hence, the current finding indicates that BDLE increases the stability of REM sleep, and that this effect does not seem to be directly related to circadian processes, but may be the result of lower pre-bedtime arousal.

These findings could also be put in context of the two process theory of sleep–wake regulation^34,35^. This model explains how both homeostatic sleep pressure (process S) and the circadian pacemaker (process C) interact to regulate sleep. It is well known that EEG delta-activity in NREM sleep increases with longer pre-sleep wakefulness in an hourglass manner, and that it decays as a function of consolidated NREM sleep. The probability of entering REM sleep increases with duration of NREM sleep through each sleep cycle, and the duration of REM sleep episodes increases with each NREM-REM-cycle, indicating that stable REM sleep may require a degree of dissipation of process S. A hypothetical increase in NREM delta-activity in BDLE, potentially caused by lower arousal at sleep onset, may possibly contribute to consolidation of REM sleep. In this framework process S dissipation may interact with a phase-advance of process C, which also increases propensity for REM sleep^24^. However, an analysis of power frequency domain is outside the scope of this paper.

Our study focuses on healthy young adults, but if the findings are confirmed, we believe there are elements of this research that will translate to clinical practice in psychiatry. Increased REM sleep is not beneficial in all circumstances, as evidenced by the high prevalence of disinhibited REM-sleep in mood disorders^10,11,17–22^. Disinhibited REM sleep has been suggested to represent a maladaptive stress response that maintains depressive episodes^10,22,23^ and impacts treatment responses^11^. However, we did not find an effect of evening BDLE on REM-density. Moreover, we did not find effects on latency to first REM-episode in our previous report on this sample^8^. These results suggest that the increased REM sleep duration in the BDLE is not a marker for disinhibited REM sleep. Rather it may be an effect of the phase-advance of circadian rhythms, potentially in combination with reduced fragmentation allowing for longer sustained episodes of REM-sleep. This may be a key finding given the potential therapeutic application of blue-depleted lighting systems in psychiatric care.

Mental disorders are also linked to fragmented REM sleep, which in combination with increased REM-density has been defined as restless REM sleep^14,16,17^. Restless REM sleep, has been found to impede overnight emotional processing, which may lead to accumulated distress, hyperarousal, and a sustaining of the restless REM sleep^15^. This mechanism has been proposed to be a transdiagnostic risk factor for mental illness, in particular insomnia, but also mood disorders. This is a different explanation of the REM sleep alterations in major depression, in that fragmented REM sleep over time leads to a shortage of time spent in REM sleep eventually resulting in a disinhibition and rebound of REM sleep in major depression^17^. The restless REM sleep hypothesis of mental illness offers another mechanism by which these findings may have beneficial effects if translated into clinical practice.

### Future directions

In the context of the restless REM sleep model of insomnia^15^, the current findings on BDLE-consolidation of REM sleep may also suggest a potential therapeutic role of evening BDLE for patients with insomnia. This may particularly be the case for the subgroup of insomnia patients reporting sleep–wake misperception, which may indicate high arousal and fragmentation in REM sleep^15^. Indeed, one small study found additional benefits of adding blue-blocking glasses to cognitive behavioral therapy for insomnia on subjective levels of anxiety and hyperarousal^36^, and another small study found effects of blue-blocking glasses on both subjective and objective sleep measures compared with clear glasses^37^. Moreover, given the rationale for restless REM sleep to exacerbate symptoms in patients with major depression and insomnia, it would also be of interest to test potential effects of BDLE on REM sleep parameters and clinical outcomes. Furthermore, other NIF-effects of evening light exposure has been found to have high interindividual variability^38^. This is likely the case for the current effects on REM sleep fragmentation (see Supplementary Fig. 8), suggesting future studies are needed to uncover the characteristics of individuals that have large effects of BDLE. Moreover, it is also conceivable that the effects of consolidating REM sleep may first be consequential if the outcome is not short-term improvement, but rather the alleviation of clinical symptoms over time or the prevention of future illness episodes. Previous case-series using extended darkness^39^ or blue-blocking glasses with patients with rapid cycling bipolar disorder found that when used consistently over years, they seemed to stabilize mood and prevent new illness episodes.

### Strengths and limitations

A strength of the current study is the use of a randomized cross-over trial design which increases power by allowing for individuals to be compared to themselves in the two LEs. Moreover, it enables inference on the causality between conditions and the outcomes. Furthermore, we had complete data on all measures. However, several limitations must be considered in the interpretation of the study findings. First, this was an opportunistic study testing hypotheses that were developed after the primary analyses were completed. The sample size was small, and the power-calculations for outcome measures in the original study does not apply to the outcome measures in these secondary analyses, increasing the probability of type II errors. Second, the participants were healthy young adult individuals that were screened for any physical illness, mental disorders, and sleep disturbance. This limits the generalizability of findings to any clinical population, and to the aged population in which photoreception may be compromised. Finally, there was only a 1-day wash-out between study periods, which increases chances for carry-over effects.

## Conclusion

This is the first study demonstrating that residing in an evening BDLE reduces the fragmentation of REM sleep and microarousals in REM sleep while REM density and N3 sleep are unaltered. Given the widespread interest in the spectral tuning of evening lighting from both hospitals, nursing homes, as well as private consumers, particular care should be taken to test these effects in larger samples. Effects should also be tested in clinical samples with clinical outcome-measures, to further understand the health consequences of the potential beneficial effects of BDLE on REM sleep fragmentation.

## Methods

The current study reports secondary analyses of data collected during a short-term (13 days) randomized cross-over trial of 12 healthy young adults. The original study protocol was approved by the Regional Ethical Committee in Trondheim (Central Norway; REK: 2017/916). The protocol was designed and the study performed in accordance with the declaration of Helsinki. The study rationale, protocol, procedures are described on the ISRCTN website (Reference 12419665) and primary findings are published elsewhere^8^. Here, we briefly summarize key information relevant to the secondary analyses. It should be noted that this is an opportunistic study (arising because of recent findings reported by other research groups), and was not planned a priori, so the analyses must be regarded as exploratory.

### Summary of the cross-over trial

**Figure 3:**
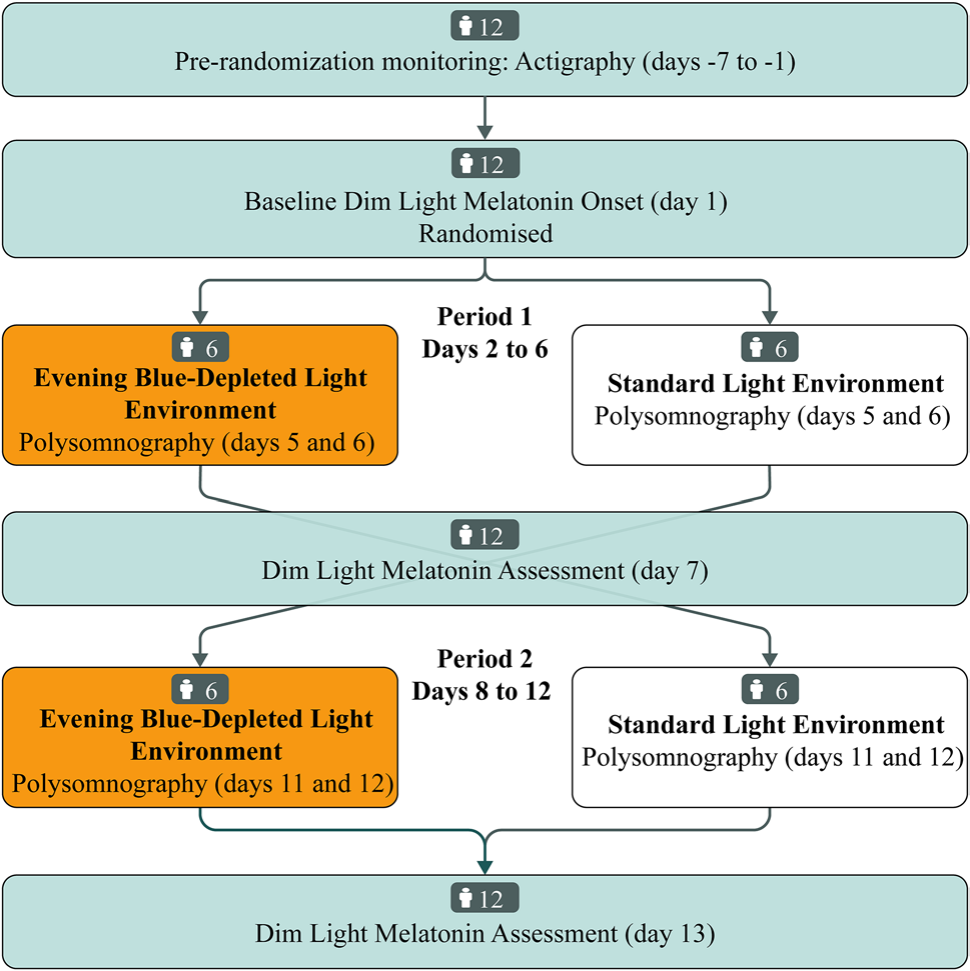
Overview of the study design, flow of participants in the study periods, and timing of relevant assessments. Adapted from the CONSORT guidelines extended to cross-over trials^43^.

Overview: The trial was undertaken between September and October 2017 and tested the effects of residing in an evening BDLE on melatonin levels, sleep, neurocognitive arousal, and subjective sleepiness. The study was undertaken in a new-build acute psychiatric unit (prior to its opening to the public) at St. Olavs Hospital in Trondheim, Norway, where dynamic light sources were installed in one of two wards. After providing written informed consent, participants underwent a 7-day pre-randomization monitoring phase. Then participants completed a 13-day protocol where DLMO was assessed on day 1, 7, and 13. On days 2–6 and 8–12, they resided for 5 + 5 days in the LEs with the order of exposure being the result of randomization (see also Fig. 3). The randomization was performed by the Unit of Applied Clinical Research (Department of Medicine and Health Sciences, NTNU).

### Participants

Healthy participants were recruited to take part in the study. Participants met inclusion criteria if their habitual sleep–wake patterns were within normal parameters (here defined as weekday bedtime between 22:30 h and 24:00 h, weekday rise time between 06:30 h and 08:00 h, and small intraindividual variations (< 2 h) between weekdays and weekends) and they tested negative on the Ishihara plate test for color blindness. Exclusion criteria were current medical or psychological conditions, current use of prescription medication(s), family history of severe mental illness, current sleep disorders, night shift work in the preceding 2 years, trans-meridian travel in the preceding 2 months exceeding one time-zone, and/or current use of nonprescription drugs or illicit substances (not including alcohol or nicotine). Moreover, for the last 7 days preceding randomization, participants were requested to maintain a fixed sleep wake-schedule (bedtimes: 23:00 h–00:00 h, risetimes: 07:00 h–08:00 h). They were further asked to refrain from ingesting alcohol and/or caffeine after 12:00 h for the duration of the study.

Daily routine: Participants were awoken at 07:00 h each morning, left the unit by 08:00 h and returned by 17:00 h for a shared meal. At 18:00 h participants entered their assigned LE and were free to spend their time in their private rooms or the common areas. Participants retired to their bedrooms for sleep by 23:00 h and turned the lights off. Hospital staff were present at all times to ensure the safety of the participants and assist in the running of the unit including meals.

### The evening light environments

*Evening BDLE:* The light environment was created using a Light Emitting Diode (LED) lighting system containing red, blue and green-white diodes that could be individually programmed to emit different colored light. From 18:30 h until 06:50 h, this system generated blue-depleted lighting in bedrooms, bathrooms, hallways and common areas using a combination of the green-white and red diodes. Blue blocking filters automatically descended to cover windows in the evening and were retracted in the morning, whereas televisions had permanent blue-blocking filters. In addition, participants were asked to use physical blue-blocking filters (lowbluelights.com) on their electronic media devices in the evenings. During daytime, all diodes were used to generate a standard white hospital light.

*Standard LE:* The light environment had standard white hospital light at all times. TV screens and other electronic devices were used as normal, without blue-blocking filters.

During the blue-depleted light period light levels (photopic lux) were similar in both wards, whereas levels of melanopic lux were lower in the evening BDLE (for detailed light measurements, see Vethe et al.^8^).

### Assessments

See also Fig. 3 for an overview of the timing of exposure to the experimental conditions and the timing of assessments.

#### Polysomnographic recordings

Polysomnographic (PSG) recordings were used to assess sleep on the last two nights (4th and 5th) in each LE. The 10–20 system for electroencephalography recording was used in the mounting of the PSG equipment and included the F3, F4, C3, C4, O1 and O2 electrodes. Electrooculogram, submental electromyogram, electrocardiogram, peripheral pulse oximetry and electrodes on the legs were also used. The SOMNO HD (SOMNOmedicsGmbH, Randersacker, Germany) PSG-equipment were used to collect the data. Signals were sampled at 256 or 128 Hz, low-pass filtered, and stored at 128 Hz. Sleep stage scoring was performed according to the American Academy of Sleep Medicine rules^40^ by a clinical neurophysiologist with > 10 years of experience with PSG-scoring who was blinded to participant details (individual characteristics, LE condition, etc.). After sleep stage scoring REM sleep epochs were visually inspected once more and all rapid eye movements (REMs) were marked by 3-s mini-epochs (using similar procedures as e.g. Lechinger et al.^11^ and Feige et al.^41^). REM sleep microarousals was scored using an automatic scoring algorithm embedded in the PSG-scoring software DOMINO. Duration-criterion for cortical arousal was set to 3 s, and arousals were required to be accompanied by an EMG increase.

#### Dim light melatonin onset assessments (DLMO)

We performed melatonin assessments to estimate the phase shift of DLMO from baseline until the first evening after having resided in the different evening LEs for 5 days. This translates into: a baseline assessment before entering the first light condition (day 1); an intermediary evening after 5 days in the first LE (day 7); and after 5 days in the second LE (day 13). Participants stayed in a dim light environment (< 3 lx) from 18:00 h until 23:00 h where saliva samples were collected every 60 min following a standard protocol^8^. DLMO was defined as the clock time when melatonin levels surpassed a 4 pg/mL threshold.

#### Pre-randomization sleep-monitoring

During the pre-randomization monitoring phase participants registered their sleep in sleep diaries upon awakening every morning, and wore an actiwatch (Actiwatch Spectrum, Philips Respironics Inc., Murrysville, PA). Participants were instructed to press an event-marker button on the actiwatch to indicate bedtime. For days where an event-marker press was missing, bedtimes indicated in the sleep diaries were used instead. Actigraphy data (sampled using 30 s epochs) was analyzed using an automatic scoring program (Actiware version 5.70.1, Philips Respironics Inc., Murrysville, PA) to calculate the rise-times and total sleep times during the pre-randomization monitoring phase.

### Statistics

We used a linear mixed model with the following variables one at a time as dependent variables: REM sleep duration, REM sleep fragmentation, REM sleep microarousals, and REM-density. The models were fitted in two steps by first entering LE and phase-shift one at a time, and then simultaneously as fixed effects. A random intercept was added for participant ID in all models. This model utilizes the cross-over design such that the effects is estimated intra-individually, resulting in higher precision than could have been achieved with a parallel group design. For the modeling of REM and N3-accumulation the combination of LE and time as percent of total sleep time rounded to the nearest integer was used as fixed effects. The difference between LEs at each percent of total sleep time was then estimated, and the resulting *p*-values were corrected for multiple comparisons using the Bonferroni correction.

Normality of residuals was checked by visual inspection of QQ-plots and with the Shapiro–Wilk test. In some analyses, there were some deviations from normality, in which case bootstrapping with 10,000 resamples were performed and bias-corrected and accelerated confidence intervals were used. Two-sided *p*-values < 0.05 were considered significant.

Statistical analyses were performed using R statistical package (version 3.6.2., https://www.R-project.org/). Linear mixed effects models were fitted (using the R-package “lme4”).

#### Post hoc analyses of order effects

To investigate if there was an effect condition order we used a linear mixed model with condition, study period number, and their interaction as fixed effects. Dependent variables were the respective REM main-outcome variables. Participant ID was entered as a random intercept.

#### Rem sleep fragmentation, REM sleep microarousals, and REM-density

REM sleep fragmentation was the total overnight number of interruptions of REM sleep by non-REM sleep or wake bouts that was occurring within REM sleep episodes^42^ divided by the total duration of REM sleep. A REM sleep episode was defined as starting on the first REM sleep epoch after a minimum of 15 min of continuous non-REM sleep and ended at the last epoch of REM sleep preceding at least 15 min of continuous non-REM sleep.

REM-density was calculated by dividing the sum duration of the 3-s mini-epochs marking REMs within REM-epochs by the total duration of REM sleep over the night^11^.

#### Phase-shift of DLMO

Phase shift of DLMO was calculated by estimating the mean change in DLMO from baseline to the DLMO-assessment after each LE. Fitted values from a linear mixed model described in the previous publication^8^ was used to calculate individual phase-shifts.

#### Accumulation of REM sleep and N3

For the accumulation of N3 and REM sleep, the epochs of the respective stages were cumulatively summed for each percent (rounded to the nearest integer) of the sleep period.

## Supplementary Information

Supplementary Information.
