# Supplementary material for: Evening light environments can be designed to consolidate and increase the duration of REM-sleep
Source: Sci Rep. 2022 May 24;12:8719. doi: 10.1038/s41598-022-12408-w (PMC9130237; doi:10.1038/s41598-022-12408-w)
Supplement: Supplementary file 1 — Supplementary Information. [file 41598_2022_12408_MOESM1_ESM.docx]

**Supplements**

*Supplementary figure S1. Boxplot indicating median duration of completed sleep cycles. Hinges indicate the first and third quartile. Whiskers extend to 1.5*the inter quartile range (distance between 1^st^ and 3^rd^ quartile).*

**

*Supplementary figure S2. Mean duration of rem episode in each sleep cycle by condition. Error bars indicate means ± SEM. Due to low N (5 nights) in cycle 6, these data-points were not included in the figure.*

**

*Supplementary figure S3. Mean duration of NREM sleep during each sleep cycle by condition. Error bars indicate means ± SEM. Due to low N (5 nights) in cycle 6, these data-points were not included in the figure.*

*Supplementary figure S4. Mean number of REM sleep interruptions in each REM sleep cycle. Error bars indicate means ± SEM. Due to low N (5 nights) in cycle 6, these data-points were not included in the figure.*

*Supplementary figure S5. Percent of REM sleep interruptions lasting longer than 3 minutes by cycle number. Error bars indicate means ± SEM. Due to low N (5 nights) in cycle 6, these data-points were not included in the figure.*

*Supplementary figure S6. Distribution of sleep stages during the interruptions from REM sleep. Due to low N (5 nights) in cycle 6, these data-points were not included in the figure.*

**

*Supplementary figure S7. Observed values of (A) REM sleep fragmentation, (B) REM sleep arousals, and (C) REM sleep duration plotted against phase shift and separated by color/shape to indicate condition.*

**

*Supplementary figure S8. Individual REM sleep fragmentation in each condition. Percent REM sleep fragmentation is the number of interruptions during REM sleep divided by total duration of REM sleep in minutes times 100. Error bars indicate estimated means ± 95% confidence intervals from the linear mixed model (N = 12). The omission of the outlier value in standard LE did not meaningfully change the results of the analysis.*

|  |  | NREM microarousals per hour | | | | |
| --- | --- | --- | --- | --- | --- | --- |
| Step |  | | Estimate | 95% CI | *p* |  |
| 1 | BDLE | | -9.50 | -19.48 to 0.49 | 0.063 |  |
|  | Phase shift | | 10.71 | -1.21 to 23.50 | 0.079 |  |
| 2 | BDLE | | -6.48 | -4.95 to 18.24 | 0.27 |  |
|  | Phase shift | | -6.36 | -33.25 to 2.83 | 0.38 |  |

*Supplementary Table S1. Results from a similar mixed model to the main analyses fitted with NREM microarousals per hour of NREM sleep as the dependent variable. Independent variables are BDLE and phase shift. In the first step variables are tested separately, and in the second step variables are entered together. N=12 participants. BDLE=Blue-depleted light environment*
